# Existing Climate Change Will Lead to Pronounced Shifts in the Diversity of Soil Prokaryotes

**DOI:** 10.1128/mSystems.00167-18

**Published:** 2018-10-23

**Authors:** Joshua Ladau, Yu Shi, Xin Jing, Jin-Sheng He, Litong Chen, Xiangui Lin, Noah Fierer, Jack A. Gilbert, Katherine S. Pollard, Haiyan Chu

**Affiliations:** a State Key Laboratory of Soil and Sustainable Agriculture, Institute of Soil Science, Chinese Academy of Sciences, Nanjing, China; b Gladstone Institutes, San Francisco, California, USA; c Department of Ecology, College of Urban and Environmental Sciences and Key Laboratory for Earth Surface Processes of the Ministry of Education, Peking University, Beijing, China; d Key Laboratory of Adaptation and Evolution of Plateau Biota, Northwest Institute of Plateau Biology, Chinese Academy of Sciences, Xining, China; e Department of Ecology and Evolutionary Biology, University of Colorado, Boulder, Colorado, USA; f Cooperative Institute for Research in Environmental Sciences, University of Colorado, Boulder, Colorado, USA; g Biosciences Division, The Microbiome Center, Argonne National Laboratory, Argonne, Illinois, USA; h Department of Surgery, University of Chicago, Chicago, Illinois, USA; i Marine Biological Laboratory, Woods Hole, Massachusetts, USA; j Division of Biostatistics and Institute for Human Genetics, University of California, San Francisco, California, USA; k Chan-Zuckerberg Biohub, San Francisco, California, USA; Florida State University

**Keywords:** soil bacterial diversity, niche modeling, climate change, microbial biogeography, biogeography, diversity, soil microbiology

## Abstract

Soil bacteria are key to ecosystem function and maintenance of soil fertility. Leveraging associations of current geographic distributions of bacteria with historic climate, we predict that soil bacterial diversity will increase across the majority (∼75%) of the Tibetan Plateau and northern North America if bacterial communities equilibrate with existing climatic conditions. This prediction is possible because the current distributions of soil bacteria have stronger correlations with climate from ∼50 years ago than with current climate. This lag is likely associated with the time it takes for soil properties to adjust to changes in climate. The predicted changes are location specific and differ across bacterial taxa, including some bacteria that are predicted to have reductions in their distributions. These findings illuminate the widespread potential of climate change to influence belowground diversity and the importance of considering bacterial communities when assessing climate impacts on terrestrial ecosystems.

**IMPORTANCE** There have been many studies highlighting how plant and animal communities lag behind climate change, causing extinction and diversity debts that will slowly be paid as communities equilibrate. By virtue of their short generation times and dispersal abilities, soil bacteria might be expected to respond to climate change quickly and to be effectively in equilibrium with current climatic conditions. We found strong evidence to the contrary in Tibet and North America. These findings could significantly improve understanding of climate impacts on soil microbial communities.

## Abstract (precis)

There have been many studies highlighting how plant and animal communities lag behind climate change, causing extinction and diversity debts that will slowly be paid as communities equilibrate. By virtue of their short generation times and dispersal abilities, soil bacteria might be expected to respond to climate change quickly and to be effectively in equilibrium with current climatic conditions. We found strong evidence to the contrary in Tibet and North America. These findings could significantly improve understanding of climate impacts on soil microbial communities.

## INTRODUCTION

Climate change is disrupting almost all ecosystems on Earth, with widespread effects on plants and animals (1, 2). Continued climate shifts are predicted to exacerbate these effects. But even if climate stabilized today, disruptions to ecosystems would continue for some time. Two examples are the extinction debts of many long-lived, slowly reproducing species whose populations will dwindle in coming years due to environmental shifts that have already occurred (3, 4) and the colonization lags of species whose ranges are in the process of moving in response to climate change (5). Terrestrial bacteria play fundamental roles in the functioning of ecosystems and the maintenance of soil fertility (6, 7). However, despite the fact that soil bacterial communities and the processes they mediate are often highly sensitive to climate (8), we have limited knowledge of the effects of climate change on the regional distributions of soil bacteria (9–12).

This study investigates the spatial and temporal extent of legacy effects among soil prokaryotes and the consequences of equilibration of soil prokaryotic distributions to contemporary climate. By virtue of their short generation times and dispersal abilities, soil microbes might be expected to respond to climate change quickly and to be effectively in equilibrium with current climatic conditions. However, legacy effects—defined here as community properties that persist after environmental change (13)—have been observed in soil microbial communities, which take up to 3 years to respond to drought and other environmental shifts (14–18). There is an indication of decadal-scale legacy effects in microbial enzyme activity as well (19). Microbial legacy effects are also known in agricultural (20) and other ecosystems (18, 19, 21). Moreover, because the distributions of soil bacteria are strongly influenced by edaphic characteristics (including soil pH and soil nutrient availability [22–24]), and because these soil properties change slowly over time, factors driving shifts in soil bacterial communities can reflect historic climate (25–29). Thus, soil bacterial communities may still be adjusting to existing climate change, and it may take years or decades for the full effects of existing climate change to become evident.

The Tibetan Plateau provides an ideal location to study legacy effects in soil microbial distributions. Because the plateau is undergoing rapid climate change (30), many of the factors that drive the distributions of soil microbes, particularly soil properties and plant communities, may still be equilibrating to the current climate. Understanding how climate change will affect Tibetan soil microbial communities is important: the plateau contains a vast soil carbon reservoir (31) that may become labile due to thawing permafrost and accelerated microbial metabolism (32, 33), and the region actively moderates climate in Asia and across the globe. Also, soil microbes on the Tibetan Plateau are exposed to particularly dry, cold conditions. It is the youngest (∼2.4 × 10^8^ years), largest (∼2.0 × 10^6^ km^2^), and highest (mean ∼4,000 m) plateau in the world. Because the Tibetan Plateau has an extreme and changing climate, we anticipated that modeling equilibration of soil microbial communities in the region to climate change would reveal potentially dramatic shifts.

To test this hypothesis, we measured bacterial and archaeal community composition in 180 nonagricultural soils from 60 locations across the plateau. We showed that the prokaryotic taxonomic distributions in these soils were closely associated with historic climate (from ∼50 years ago), even after adjusting for contemporary climate. Using models of associations between current prokaryotic communities and historic environmental factors, we predicted that diversity, community structure, and biogeographic patterns would shift substantially with equilibration to contemporary climate. To explore how generally applicable these findings are, we performed analogous analyses with 84 surface soil samples from across the United States and Canada. Our results suggest widespread increases in soil prokaryotic diversity in both regions and region-specific shifts in the distributions of individual taxa if these communities were to equilibrate to current climate conditions.

## RESULTS AND DISCUSSION

### Disequilibrium of prokaryotic communities with current climate.

We profiled bacterial and archaeal community structure using 16S rRNA gene amplicon sequencing from 180 surface soil samples across 60 locations in the Tibetan Plateau and obtained a total of 926,609 reads (median = 5,247 per sample, range = 3,016 to 9,926 per sample). These communities had 65,874 operational taxonomic units (OTUs) and were dominated by nine phyla (see Fig. S1D and Table S1A in the supplemental material).

10.1128/mSystems.00167-18.1 FIG S1 Overview of community composition, sampling sites, and climate data in the Tibetan Plateau. (A to C) Prokaryote community compositional structure in the Tibetan Plateau soils as indicated by nonmetric multidimensional scaling plots. Sites are color coded according to soil moisture content. (A) Based on Bray-Curtis distance. (B) Based on unweighted UniFrac distance. (C) Based on weighted UniFrac distance. (D) Relative abundance of the dominant phyla across samples from Tibetan Plateau. Relative abundances are based on the proportional frequencies of 16S rRNA sequences that could be classified at the phylum level. (E) Locations of sampling sites across Tibetan Plateau. (F) Principal components of climate data as a function of time show consistent trends or consistent trends followed by leveling off. There is one line for each grid cell in the climate data map that harbors one or more samples. Download FIG S1, PDF file, 0.2 MB. Copyright © 2018 Ladau et al. 2018 Ladau et al. This content is distributed under the terms of the Creative Commons Attribution 4.0 International license.

10.1128/mSystems.00167-18.10 TABLE S1 (A) Relative average abundances of phyla classified with the Greengenes database (http://greengenes.lbl.gov) across all soils. Column headers are sample names. (B) The shifts in relative abundance of the bacterial families (Tibetan Plateau). Column headers are defined in an adjacent sheet in the Excel file. (C) The shifts in relative abundance of the bacterial families (North American). Column headers are identically defined as for Table S1B (see column definition sheet). (D) Correlations of richness and phylogenetic diversity (PD) with soil characteristics, plant Shannon index, and plant species richness. (E) Soil variables significantly correlated with structure of prokaryote communities, as determined by Mantel tests. (F) Description of the geographic, climatic, and soil variables by study sites. Column headers are defined in an adjacent sheet in the Excel file. (G) Climate variables used in modeling. Download Table S1, XLSX file, 0.3 MB. Copyright © 2018 Ladau et al. 2018 Ladau et al. This content is distributed under the terms of the Creative Commons Attribution 4.0 International license.

We obtained monthly maps of 10 climate variables across the plateau at 0.5-degree resolution from 1950 to 2012 (34). To dampen noise from short-term fluctuations, for each climate variable, we created climatologies by averaging values at our sampling locations over 10-year sliding windows. Results from 20-year climatologies were qualitatively similar. We refer to the 10-year climatologies by the dates that they span (e.g., 1950–1959 climatology averages climate data from 1950 to 1959). We also calculated 1-year climatologies from the year when samples were collected to account for effects of contemporary climate. We performed principal component analysis (PCA) jointly on all the climatologies (historical and current) and found that the first three principal components cumulatively account for 88.1% of variation (54.4% PC1, 23.3% PC2, and 10.4% PC3) while reducing its dimensionality, which is key for model selection in the following analyses. For most time periods, temperature mean is highly weighted in PC1, precipitation mean in PC2, and temperature range in PC3. In order to make all-subset model selection computationally feasible, we use the projections onto the first three principal components (three values for each time period and geographic location) in lieu of the full climate data matrix (ten values for each time period and geographic location), referring to these PCA-based summaries of the climatologies as “climate variables.”

To assess whether the soil prokaryotic communities are in equilibrium with contemporary climate in the Tibetan Plateau, we built a regression model of OTU richness (number of OTUs) as a function of historical and contemporary climate variables. By performing all-subset model selection in which climate variables from different time periods compete with each other based on how well they can explain variation in OTU richness across sampling locations, we assessed the extent to which the contemporary distribution of prokaryotic diversity is associated with historic and contemporary climate (Materials and Methods). We also performed analogous analyses to assess correlations of contemporary and historical climate with Shannon diversity (evenness of OTUs) and with relative abundance of each prevalent bacterial family (*n* = 53) and OTU (*n* = 317) found in 40 or more soil samples. No archaea met this prevalence threshold.

**FIG 1:**
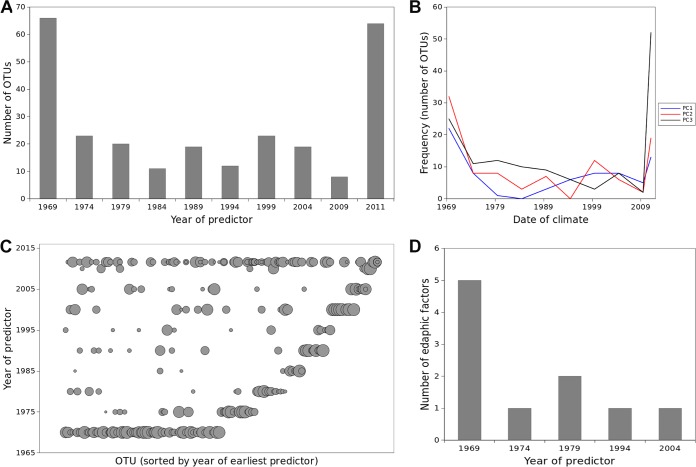
Distributions of soil prokaryotes in Tibet lag behind shifts in climate by up to 50 years. (A) The number of OTUs associated with climate from different years. A given OTU can be associated with climate from multiple years; the 2011 category represents climate from the year of sample collection. Lags are indicated by the association of many OTUs with climate from prior to 2011 and in many cases prior to 1980. (B) OTUs were associated with climate from both contemporary and historic values of most climate variables (PC1, PC2, and PC3 are associated with temperature, precipitation, and temperature range, respectively). (C) Most OTUs associated with historic climate were also associated with contemporary climate. Symbol size is proportional to the strength of the association, and OTUs (*x* axis) are ordered by the earliest year of climate with which they were associated. (D) Soil properties were also associated with historic climate, suggesting that the lags in bacterial distributions may be mediated by or associated with lags in soil properties. Climate from all time periods competed in the model selection procedure for each edaphic factor, and only the resulting predictive associations are included in the histogram.

Soil prokaryotic distributions that are significantly correlated with the climate from several decades ago as opposed to the climate from the time of sampling could be explained by distributions that are out of equilibrium with contemporary climate, among other potential contributing forces (see below). Consistent with this, climate from before 1974 predicted contemporary prokaryotic richness (i.e., was frequently chosen over many iterations of model selection in models with different numbers of variables as quantified by the Lindeman, Merenda, and Gold statistic [LMG] [35]): 1960–1969 PC1 LMG = 0.183, 1960–1969 PC3 LMG = 0.202. Contemporary climate variables were also predictive: 2002–2011 PC2 LMG = 0.415, 2002–2011 PC3 LMG = 0.200. In contrast, intervening years’ climatologies were less often chosen during model selection. Contemporary Shannon diversity is highly correlated with richness and hence is also predicted by both historic and contemporary climate. For models to predict the relative abundance of prevalent families and OTUs, the importance of climate across the decades spanning 1959 to 2012 was substantial and consistent (Fig. S2A and S3A). However, the frequency with which climate variables from different decades were predictive was bimodal: both historic variables from circa 1969 and contemporary variables were frequently predictive of the distributions of families and OTUs, while variables from circa 1980 were less frequently predictive (i.e., less often chosen in model selection) (Fig. 1A and Fig. S2B). This bimodality held quite generally across different climate variables for both OTUs (Fig. 1B) and families (Fig. S2C). Furthermore, contemporary distributions of families and OTUs were often simultaneously associated with both historic and contemporary climate (Fig. 1C and Fig. S2D). These results suggest that contemporary distributions of the diversity of soil prokaryotes and of individual taxa are associated with climate from today and from close to 50 years ago, or potentially earlier, as our results cannot rule out effects from before the time period we investigated.

**FIG 2:**
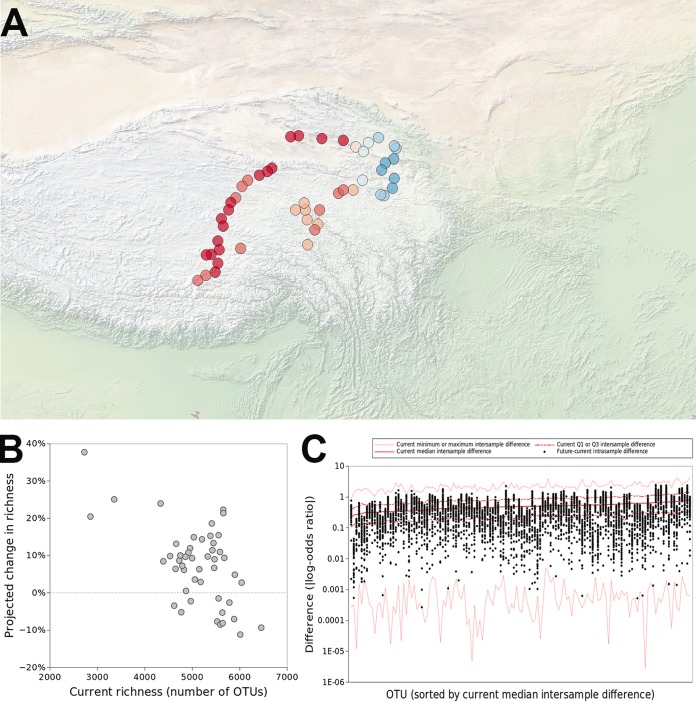
With equilibration to contemporary climate, the distributions of soil prokaryotes in Tibet would shift substantially. (A) Across most of the locations sampled, richness would increase, although in some locations it would decrease. Red and blue indicate increases and decreases in richness, respectively. (B) Increases in richness would be greatest in locations that have relatively low richness; locations with higher contemporary richness would see little change, or even decreases in richness. (C) The magnitude of shifts in relative abundance of OTUs with equilibration would be comparable to contemporary intersample variability in their relative abundance. Red lines indicate current intrasample differences in relative abundance; black dots represent the projected shifts in relative abundance with equilibration.

10.1128/mSystems.00167-18.2 FIG S2 Results for soil bacterial families across the Tibetan Plateau. Abundances of 53 prevalent bacterial families (>40 samples) are associated with historical climate. (A) Importance of climate variables for predicting distributions of families. Historic climate variables tend to be as important as contemporary climate variables. (B to D) Same as Fig. 1A to C, but for families. (E) Same as Fig. 2C, but showing the distribution of current intersample differences in relative abundances of families. (F) Same as Fig. 2C, but showing the distribution of projected future intersample differences of relative abundances of families. (G) Shifts in the relative abundance of families as a function of their current relative abundance. Red, increases; blue, decreases. Shifts are significantly inversely associated with current relative abundance, as evidenced by blue symbols being clustered in the upper part of the graph. (H) Same as Fig. 2C, but showing the distribution of projected future intersample differences in relative abundances of families. Download FIG S2, PDF file, 0.3 MB. Copyright © 2018 Ladau et al. 2018 Ladau et al. This content is distributed under the terms of the Creative Commons Attribution 4.0 International license.

10.1128/mSystems.00167-18.3 FIG S3 Results for soil bacterial OTUs across the Tibetan Plateau. Abundances of 317 prevalent bacterial OTUs (present in >40 samples) are associated with historical climate. (A) Same as Fig. S2, but for OTUs. (B) Analogous to Fig. 2C, but showing the distribution of current intersample differences in relative abundances of OTUs. (C) Same as Fig. S2G, but for OTUs. (D) Analogous to Fig. 2C, but showing the distribution of projected future intersample differences in relative abundances of OTUs. Download FIG S3, PDF file, 0.3 MB. Copyright © 2018 Ladau et al. 2018 Ladau et al. This content is distributed under the terms of the Creative Commons Attribution 4.0 International license.

Historical climate may be an important predictor of contemporary prokaryotic distributions because soil edaphic characteristics often follow historic conditions (36). Indeed, we applied the same modeling procedure used for family/OTU abundance to predict current soil characteristics as a function of climate, allowing climatologies from different decades to compete in the model selection procedure. We found that historical climate variables were more predictive than current climate of soil edaphic characteristics (Fig. 1D) and that five key soil edaphic characteristics (dissolved organic nitrogen, soil organic carbon, total carbon, dissolved organic carbon, and total nitrogen) were particularly strongly correlated with climate from before 1980 (Fig. S4A). Even though most soil microbes likely have short generation times, the diversity and composition of soil microbial communities appear to be strongly influenced by soil properties that change slowly over time (37). Like the microbial communities, these soil properties are out of equilibrium with contemporary climate.

10.1128/mSystems.00167-18.4 FIG S4 Results for soil properties across the Tibetan Plateau. (A) Same as Fig. 1C, but for soil properties. DON, dissolved organic nitrogen; NO_3_, nitrate nitrogen; DOC, dissolved organic carbon; CN ratio, carbon/nitrogen ratio; DTN, dissolved total nitrogen. (B) Relationship between relative abundance of dominant bacterial phyla and soil C:N ratio. Linear regressions were used to test Pearson correlation between the relative abundance of each taxon and soil C:N ratio. (C) Relationship between relative abundance of dominant bacterial phyla and soil moisture (SM). Linear regressions were used to test Pearson correlation between each taxon’s relative abundance and SM. Download FIG S4, PDF file, 0.3 MB. Copyright © 2018 Ladau et al. 2018 Ladau et al. This content is distributed under the terms of the Creative Commons Attribution 4.0 International license.

We next considered several factors that could have biased our results. We concluded that our findings cannot be explained by climate cycling, because most climate variables have trended consistently over this period (Fig. S1F). Moreover, anthropogenic impacts other than climate change (e.g., land use change or pollution) are unlikely to have generated the disequilibria because the surface samples we analyzed are from undisturbed soils and anthropogenic impacts cannot account for the associations between current prokaryotic distributions and historic climatic conditions that persist even after the associations with current climate have been taken into account. However, because we considered only climate postdating 1950, we cannot exclude the possibility that climates from prior to 1950 are predictive or that legacy effects are longer than 50 years. Finally, we expect our results to be robust to the fact that soil communities were sampled at only one time during the year based on prior literature on this topic (38), although we cannot rule out the possibility that year-round sampling would change some of our findings.

To evaluate the extent to which our findings might be influenced by modeling choices, we conducted two robustness analyses. First, we performed regression modeling with the original climate variables rather than projections onto principal components, using a modified model selection procedure because all-subset selection is computationally prohibitive on 120 climatologies (10 variables × 12 time periods per location; see Materials and Methods). Second, we repeated our investigation of the association between climate and prokaryotic distributions using gradient boosting rather than standard regression. Conclusions from both of these alternative approaches were highly concordant with our primary findings, indicating that our results are not artifacts of a particular model or way to quantify climate data.

### Widespread shifts in distributions of Tibetan soil bacteria and archaea.

We next asked whether soil prokaryotic diversity would increase or decrease as communities equilibrated to current climatic conditions. To answer this question, we projected the models, which were fitted with historical and contemporary climate data, to contemporary climate data. Specifically, by inputting 2002–2011 climatology data into the models with best performance (e.g., models utilizing 1960–1969 PC1 and PC3), we forecast how prokaryotic diversity and relative abundance would change if the distributions of bacteria and archaea were to equilibrate to 2002–2011 climatology. To understand the extent of these changes, we sought to answer four specific questions. (i) With equilibration, would diversity and relative abundance predominantly increase, decrease, or remain unchanged across the sampling locations? (ii) How would shifts in diversity and relative abundance compare to existing spatial variation in diversity and relative abundance? (iii) Would locations with currently high diversity or relative abundance experience different changes compared to the locations with low diversity and low relative abundance (i.e., “rich get richer” versus homogenization)? (iv) Would intersample variability increase or decrease in the future?

We forecast that (i) richness and Shannon diversity would increase across 75% and 72.9% of the sampling locations, respectively, with an average magnitude of +7.5% (standard error, 1.5%) for richness and +2.1% (standard error, 0.4%) for Shannon diversity (Fig. 2A). We further forecast (ii) that shifts in diversity within samples would be of similar magnitude to existing intersample differences in diversity, suggesting major, although not unprecedented, shifts in diversity (Fig. S5A and B). We forecast that (iii) locations with low diversity would experience the largest increases in diversity, and locations with high diversity would experience little or no increase in diversity (Fig. 2B and Fig. S5C). The latter trend might suggest that intersample variation in diversity levels would decrease in the future, and our forecasts do indicate (iv) a moderate decrease in intersample variability in their diversity (Fig. S5A and B; differences in diversity calculated as percent changes between samples or within samples at different time points). These predictions suggest major changes in the spatial distribution of diversity in soil prokaryotic communities across the Tibetan Plateau with equilibration to existing climate change.

10.1128/mSystems.00167-18.5 FIG S5 Results for diversity in the Tibetan Plateau. (A) Distributions of projected shifts in prokaryote richness within samples compared to current and future intersample differences in richness. Projected intrasample changes in richness are of similar magnitude to existing and projected future intersample differences in richness. (B) Same as panel A, but for Shannon diversity. (C) Same as Fig. 2B, but for Shannon diversity. (D) Amount of extrapolation necessary to make geographic projections of diversity. Maps show multivariate environmental similarity surface (MESS) values, which give how far out of the observed range climate conditions are at each location. Almost all locations within the Tibetan Plateau are less than 20% out of range (greater than −20 on the map) for both current and future projections, indicating that to make geographic projections, minimal extrapolation beyond the range of the observed data is necessary. Download FIG S5, PDF file, 0.2 MB. Copyright © 2018 Ladau et al. 2018 Ladau et al. This content is distributed under the terms of the Creative Commons Attribution 4.0 International license.

**FIG 3:**
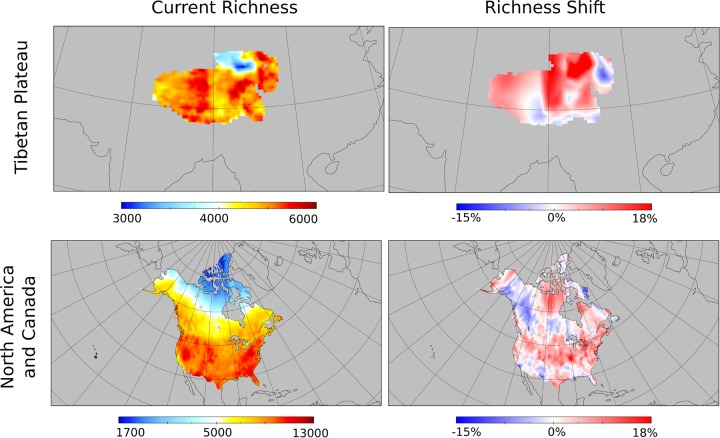
Model predictions in the Tibetan Plateau and North America show increasing prokaryotic diversity in both regions. Models of prokaryotic richness fitted on data from sampling locations were used to predict current richness across the Tibetan Plateau (top left) and northern North America (bottom left). Then the same models were used to predict future richness by plugging in current climate data. When these forecasted maps were compared to the current maps, they revealed large shifts in richness in both regions (right), with richness increasing in the majority of locations (red) but decreasing in others (blue).

Further supporting this contention, we used the regression models developed above to forecast how richness would shift if prokaryotic distributions were to equilibrate across the Tibetan Plateau. Specifically, we projected maps of current richness using the historic and contemporary climate conditions that were selected for the models. Using just contemporary climate conditions, we then projected richness maps if prokaryotic distributions were to equilibrate to contemporary climate. These projections (Fig. 3) were consistent with foregoing results, suggesting widespread increases in richness across Tibetan Plateau.

Turning to how the relative abundance of individual prevalent prokaryotes might respond to equilibration to current climate, we predicted that (i) that different bacterial taxa would respond heterogeneously: some would increase across all sampling locations, but others would decrease across all sampling locations; the magnitude of changes ranged from nonsignificant to over 100% for different taxa (Table S1B). For example, *Mycobacteriaceae* and *Rubrobacteraceae* (families of *Actinobacteria*), *Bacillaceae* (a family of *Firmicutes*), and *Rhodobiaceae* and *Rhizobiaceae* (families of *Alphaproteobacteria*) are predicted to increase consistently in most locations of the plateau, while *Flavobacteriaceae* (a family of *Bacteroidetes*), *Nakamurellaceae*, and *Nocardiaceae* (families of *Actinobacteria*) are predicted to decrease in the majority of locations, albeit by modest amounts (Table S1C). Overall, there was no consistent trend across all families or OTUs (e.g., most families increasing). However, (ii) shifts in relative abundance across taxa would consistently be of similar magnitude as existing intersample differences in relative abundance (Fig. 2C and Fig. S2E and F and S3B). That is, the projected changes in community composition are on par with existing intersite variation in communities across the sampled locations. Furthermore, (iii) locations with low relative abundance would experience larger changes than locations with high relative abundance (Fig. S2G and S3C). Finally, although the latter shifts could act to even out the spatial distribution of relative abundance, this does not appear to be the case: (iv) with equilibration, intersample differences in relative abundance would be similar to contemporary intersample differences (Fig. S2H and S3D). Thus, across the Tibetan Plateau, our models predict that different taxa would undergo varying shifts in relative abundance with equilibration.

Our forecasts of shifts with equilibration to existing climate assume temporal niche conservatism, which means that bacteria and archaea are associated similarly with environmental conditions over time, as they equilibrate to changes. We also assume that the taxa we detected in each region will move into new locations or out of current locations (and similarly, alter their relative abundances at different locations) in accordance with their niche preferences, which of course depend upon dispersal. Prokaryotes could shift to occupy different niches in the time that it would take their distributions to equilibrate to contemporary climate. Such shifts would depend on rates of adaptation, dispersal, and population growth, among other factors. Incorporating these forces into our models would introduce substantial complexity and numerous assumptions. Thus, our forecasts can be taken as baseline estimates: future analyses that incorporate additional complexity, including evolution, dispersal limitation, and neutral assembly processes, may add to these results.

### Increases in diversity of northern North American bacteria and archaea.

**FIG 4:**
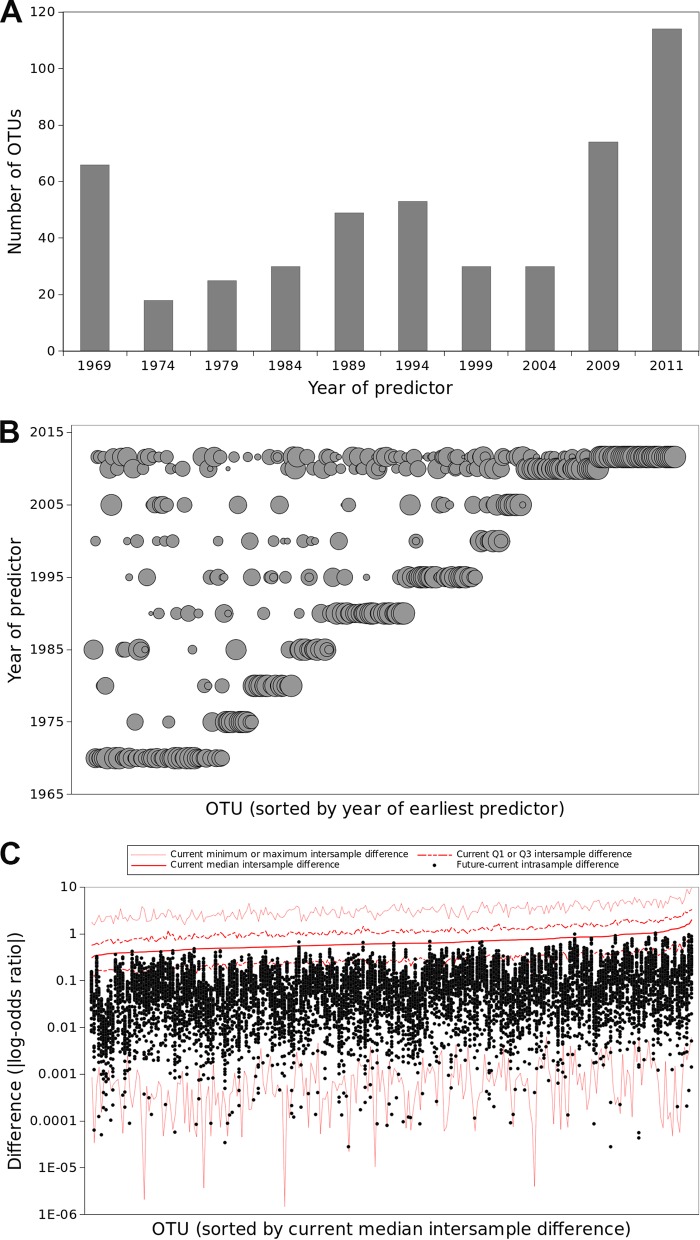
Associations between taxa and climate over time in North America. (A) The number of OTUs associated with climate from different years. (B) Most OTUs associated with historic climate were also associated with contemporary climate. Symbol size is proportional to the strength of the association, and OTUs (*x* axis) are ordered by the earliest year of climate with which they were associated. (C) The magnitude of shifts in relative abundance of OTUs with equilibration would be comparable to contemporary intersample variability in their relative abundance. Red lines indicate current intrasample differences in relative abundance; black dots represent the projected shifts in relative abundance with equilibration.

To assess whether the predicted responses of Tibetan Plateau soils to climate change are similar to those for other regions of Earth and at larger spatial scales, we performed similar analyses using historic climate data and published surface soil prokaryotic community data from 84 locations across northern North America (Fig. S6A) (24, 39). Several of the major trends from the Tibetan Plateau were also observed in northern North America. First, historical climate variables were strong predictors of taxon abundance and diversity metrics. For instance, soil prokaryotic richness was predicted by 1960–1969 climatologies in both regions (in northern North America, LMG for 1960–1969 PC2 and PC3 0.057 and 0.227, respectively, with LMG for PC1 in 1975–1984 = 0.716). Second, across families and OTUs, relative abundances were commonly predicted by both historical and contemporary climate in northern North America (Fig. 4A and B; Fig. S7A and B). When they were included via model selection, historical climate variables were important predictors (Fig. S7C and S8A). Furthermore, historical values of most climate variables were predictive (Fig. S7D and S8B). Finally, when these models were projected to contemporary climate, the forecast outcomes of equilibration were similar to those in the Tibetan Plateau: richness and Shannon diversity would increase across 76.0% and 73.0% of samples, respectively (Fig. S6B). Projecting maps of the increases in richness, these increases in richness would be geographically widespread (Fig. 3). Projected shifts in diversity and OTU relative abundance are within the range of current intersample differences in these quantities, while projected shifts in family relative abundance are mostly lower than current intersample differences (Fig. 4C; Fig. S7E and F, S8C, and S9A and B).

10.1128/mSystems.00167-18.6 FIG S6 Maps for northern North America. (A) Locations of sampling sites. (B) Analogous to Fig. 2A, but for richness. Download FIG S6, PDF file, 0.2 MB. Copyright © 2018 Ladau et al. 2018 Ladau et al. This content is distributed under the terms of the Creative Commons Attribution 4.0 International license.

10.1128/mSystems.00167-18.7 FIG S7 Results for soil bacterial families in northern North America. (A) Analogous to Fig. 4A, but for families. (B) Analogous to Fig. 4B, but for families. (C) Analogous to Fig. S2A, but for families across North America. (D) Analogous to Fig. 1B, but for families across North America. (E) Analogous to Fig. 2C, but showing the distribution of current intersample differences in relative abundances of families across northern North America. (F) Projected shifts in bacterial family relative abundances, analogous to Fig. 2C, but for families across North America. (G) Analogous to Fig. S2G, but for families across North America. (H) Analogous to Fig. 2C, but showing the distribution of predicted future intersample differences in relative abundances of families across northern North America. Download FIG S7, PDF file, 0.4 MB. Copyright © 2018 Ladau et al. 2018 Ladau et al. This content is distributed under the terms of the Creative Commons Attribution 4.0 International license.

10.1128/mSystems.00167-18.8 FIG S8 Results for soil OTUs across northern North America. (A) Analogous to Fig. S2A, but for OTUs across North America. (B) Analogous to Fig. 1B, but for OTUs in North America. (C) Analogous to Fig. 2C, but showing the distribution of current intersample differences in relative abundances of OTUs across North America. (D) Analogous to Fig. S2G, but for OTUs across North America. (E) Analogous to Fig. 2C, but showing the distribution of predicted future intersample differences in relative abundances of OTUs across North America. Download FIG S8, PDF file, 0.4 MB. Copyright © 2018 Ladau et al. 2018 Ladau et al. This content is distributed under the terms of the Creative Commons Attribution 4.0 International license.

10.1128/mSystems.00167-18.9 FIG S9 Results for diversity across northern North America. (A) Analogous to Fig. S5A, but for richness across North America. (B) Analogous to Fig. S5A, but for Shannon diversity across North America. (C) Analogous to Fig. 2B, but for richness across North America. (D) Analogous to Fig. 2B, but for Shannon diversity across North America. (E) Amount of extrapolation necessary to make geographic projections of diversity. The maps show multivariate environmental similarity surface values (MESS values), which give how far out of the observed range climate conditions are at each location. Almost all locations within North America are less than 20% out of range (greater than −20 on the map) for both current and future projections, indicating that to make geographic projections, minimal extrapolation beyond the range of the observed data is necessary. Download FIG S9, PDF file, 0.3 MB. Copyright © 2018 Ladau et al. 2018 Ladau et al. This content is distributed under the terms of the Creative Commons Attribution 4.0 International license.

Despite these similarities, we observed several important differences between our models for northern North America and the Tibetan Plateau. In northern North America, changes in richness after equilibration to current climate would be uncorrelated with current richness (Fig. S9C), although changes in Shannon diversity would be negatively associated with current Shannon diversity (Fig. S9D). Furthermore, prevalent bacterial families and OTUs would generally have the greatest changes in relative abundance in locations where they are currently rare (Fig. S7G and S8D), although the distribution of future intersample differences in relative abundance is similar to current intersample differences (Fig. S7H and S8E), suggesting that overall many pairs of samples will maintain greater difference in diversity than forecasted for the Tibetan Plateau, where some moderate homogenization of diversity is predicted. This may be due in part to the fact that the magnitude of predicted diversity changes is much larger for low-diversity sites in the Tibetan Plateau (Fig. 2B) than for North America (Fig. S9C). The most striking difference between the regions is that individual taxa have very different forecast changes in their distributions (*R*^2^ = 0.053 for correlation, between regions, of fraction of locations where families would increase). For example, *Beijerinckiaceae* (a family of *Alphaproteobacteria*) and *Acidobacteriaceae* (a family of *Acidobacteria*) are predicted to increase in relative abundance in most locations of northern North America (Table S1C), while *Methylobacteriaceae* (a family of *Alphaproteobacteria*) and *Cellulomonadaceae* (a family of *Actinobacteria*) are predicted to decrease. Thus, our results demonstrate different responses among specific bacterial taxa and between Tibetan Plateau and North America despite parallel trends toward higher diversity.

### Proximate causes of the disequilibrium.

**FIG 5:**
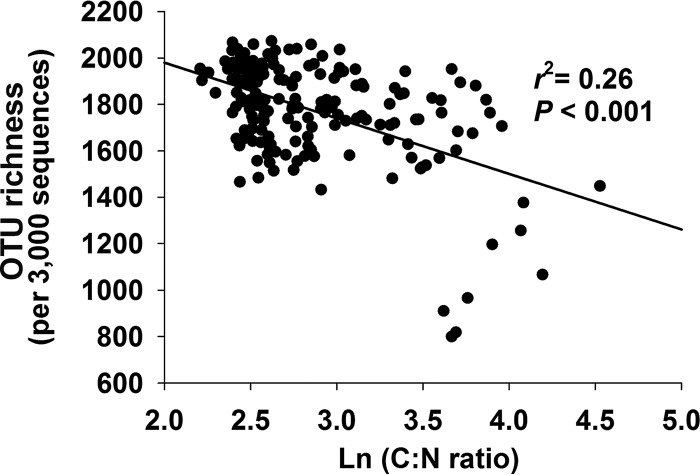
Relationship between OTU richness and soil C:N ratios.

**FIG 6:**
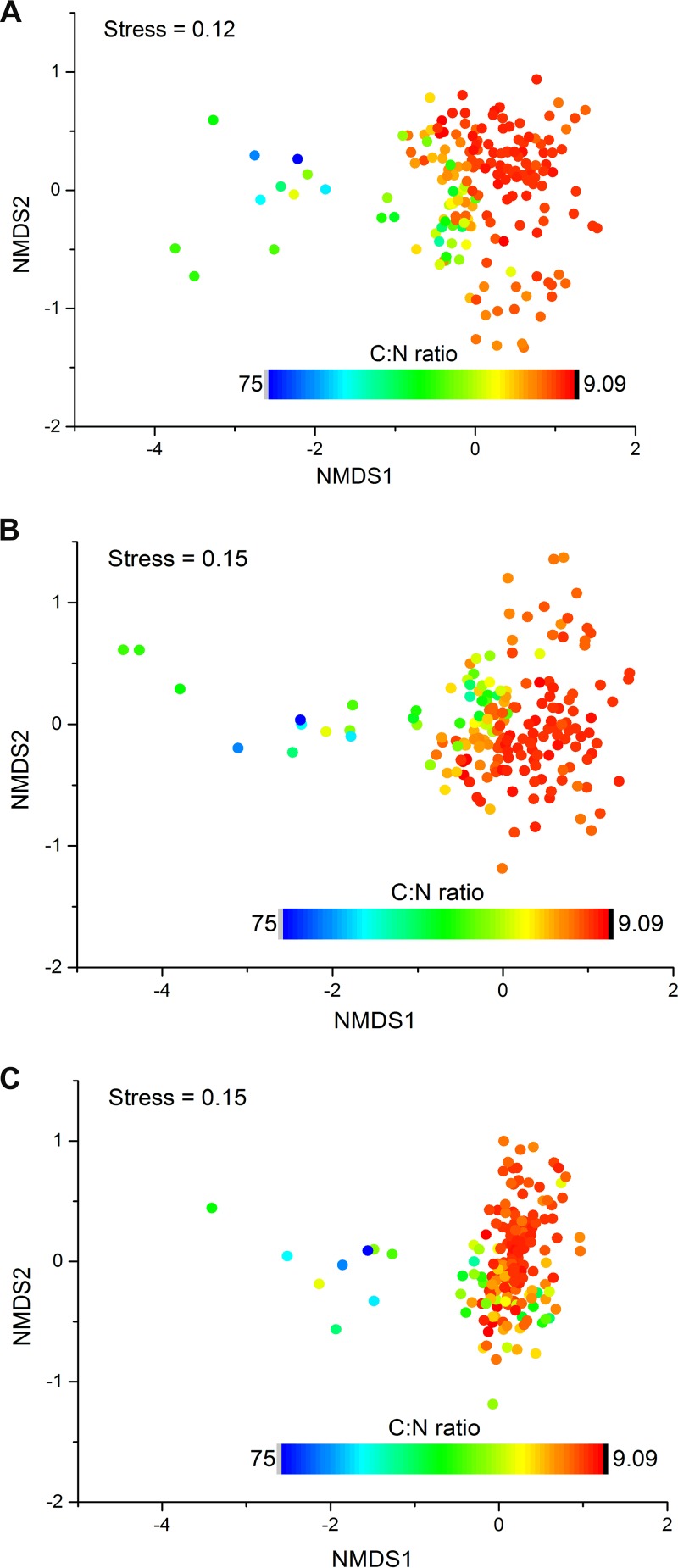
Prokaryotic communities in Tibetan Plateau soils are associated strongly with soil C:N ratios. Prokaryotic community compositional structure in the Tibetan Plateau soils as indicated by nonmetric multidimensional scaling plots. Sites are color coded according to soil C:N ratios. (A) Based on Bray-Curtis distance. (B) Based on unweighted UniFrac distance. (C) Based on weighted UniFrac distance.

The disequilibrium between prokaryotic distributions and contemporary climate is likely due to the soil properties being out of equilibrium with contemporary climate, which occurs for a variety of reasons (40). To explore this hypothesis, we analyzed correlations of current surface soil properties with patterns of prokaryotic diversity at the locations that we sampled in the Tibetan Plateau. Soil factors, such as dissolved organic nitrogen (DON) and carbon-to-nitrogen ratio (C:N), were significantly correlated with OTU richness, while soil moisture, C:N, pH, and various forms of both nitrogen and carbon were significantly correlated with community structure (false discovery rate < 0.05; Table S1D and E). In some other studies at this scale, pH had a strong association with soil prokaryotic richness (41), particularly in acidic soils (42) and when a wide range of pH values is observed. However, at sampling locations in this study, the soil C:N was more important, being negatively correlated with richness (*r*^2^ = 0. 26, *P* < 0.001) (Fig. 5 and Table S1D) and community structure (Bray-Curtis dissimilarity; *r* = 0.44, *P* = 0.001) (Fig. 6 and Table S1E). C:N is also the best predictor of the relative abundance of some, but not all, individual taxa. The high altitude and low temperatures on the plateau reduce C degradation rates and lead to N limitation (31), resulting in elevated C:N ratios and high inorganic C in dry areas. Soil moisture, which correlated with C:N, showed similar associations with richness and community composition (Fig. S1A, B, and C and Table S1D). The relative abundances of specific taxa have both negative (e.g., *Alphaproteobacteria*) and positive (e.g., *Bacteroidetes*) correlations with C:N ratio and soil moisture (Fig. S4B and C). We found that C:N ratios are more closely associated with historical rather than contemporary climate (climatology of strongest association: 1960–1969), suggesting a mechanism through which prokaryotic distributions are out of equilibrium with contemporary climate: distributions of soil properties lag behind shifts in climate, which in turn cause the distributions of bacteria and archaea to lag.

To explore associations between vegetation and soil prokaryotic communities, we recorded all plants in plots adjacent to each sampling location and tested for associations between each plant and prokaryotic diversity and abundance statistics. Unfortunately, very few plant species were prevalent enough to perform this testing or predictive modeling. Analysis of the prevalent plants did not identify significant associations, suggesting that current plants are not drivers of current prokaryotic communities or that we lacked power to detect such associations. We also are unable to test for associations with historic plant distributions, since we lack these data. Hence, our results collectively suggest a relationship between prokaryotic distributions and specific soil properties, but not current vegetation. But we cannot rule out the possibility that lagging distributions of vegetation directly contribute to the lagged relationship of prokaryotes to climate and/or affect soil properties that then shape prokaryotic distributions. Future experimental or longitudinal studies could explore these questions and also investigate whether bacteria and archaea are inherently slow to respond to climate change, irrespective of changes to soil and plants.

### Conclusions.

Soil prokaryotes appear to follow soil characteristics in showing a significant lagged response to a changing climate across many decades, a pattern evident across both the Tibetan Plateau and northern North American. If bacteria and archaea could equilibrate to existing climate change, our models predict that widespread increases in diversity and shifts in community composition would occur. While it is tempting to speculate about broader impacts of these changes, inferring the functional consequences of soil microbial community differences is challenging (43). Similar to the extinction debts and colonization lags of macroorganisms (3, 5), further climate change may drive further changes in soil microbial communities.

## MATERIALS AND METHODS

### Sample collection (Tibetan Plateau samples).

To survey current bacterial and archaeal distributions across the Tibetan Plateau, we collected 180 surface soil samples from 60 sites throughout the Tibetan Plateau during the growing season (July to September) of 2011. At each site, we sampled three plots 40 m apart, and collected 5 to 7 cores per plot at a depth of 0 to 5 cm, which were subsequently combined. Our sampling locations covered more than 1,000,000 km^2^ (see Fig. S1E and Table S1F in the supplemental material) and all of the major climate zones and grassland types across the Tibetan Plateau (Table S1F). All soil samples were delivered by cooler equipped with ice packs (4°C) to the laboratory as quickly as possible, where they were stored at −20°C until processing. In addition, all vegetation in three plots (1 × 1 m^2^ or 0.5 × 0.5 m^2^) 10 m away from the soil sampling-plot was recorded and harvested to measure aboveground biomass. At each site, one soil pit was excavated to collect samples for analyses of bulk density. From this pit, three replicate soil samples were collected at a depth of 0 to 5 cm. Bulk density was obtained using a standard container with 100 cm^3^ (50.46 mm in diameter and 50 mm in height) and measured to the nearest 0.1 g.

### Soil characteristics (Tibetan Plateau samples).

Surface soil samples for C and N analyses were air-dried, sieved (2-mm mesh), handpicked to remove fine roots, and ground. Total soil C and N contents for each plot were determined by combustion (2400 II CHNS/0 Elemental Analyzer, Perkin-Elmer, Boston, MA, USA). Soil moisture was measured gravimetrically after a 10-h desiccation at 105°C. Soil pH was determined separately on each plot at each site with a fresh soil-to-water ratio of 1:5 by pH monitor (Thermo Orion-868). Bulk density was calculated as the ratio of the oven-dry soil mass to the container volume. Dissolved organic carbon, dissolved total nitrogen (DTN), ammonium nitrogen (NH_4_^+^-N), and nitrate nitrogen (NO_3_^−^-N) were determined as described previously (44).

### Molecular analyses (Tibetan Plateau samples).

Total nucleic acids from each plot were extracted from 0.5 g of soil using a FastDNA Spin kit (Bio 101, Carlsbad, CA, USA), according to the manufacturer’s instructions, and stored at −40°C. Extracted DNA was diluted to approximately 25 ng/μl with distilled water and stored at −20°C until use. A 2-μl diluted DNA sample of each plot was used as the template for amplification. The V4-V5 hypervariable regions of 16S rRNAs (Escherichia coli positions 515 to 907) were amplified using the primer set F515 (GTGCCAGCMGCCGCGG) with the Roche 454 A pyrosequencing adapter and a unique 7-bp barcode sequence, and primer R907 (CCGTCAATTCMTTTRAGTTT) with the Roche 454 B sequencing adapter at the 5′ end of each primer, respectively. Each sample was amplified in triplicate with a 50-μl reaction mixture under the following conditions: 30 cycles of denaturation at 94°C for 30 s, annealing at 55°C for 30 s, and extension at 72°C for 30 s, with a final extension at 72°C for 10 min. PCR products from each sample were pooled and purified with an agarose gel DNA purification kit (TaKaRa), combined in equimolar ratios in a single tube, and run on a Roche FLX454 pyrosequencing machine (Roche Diagnostics Corporation, Branford, CT), producing reads from the forward direction F515.

### Bioinformatics (Tibetan Plateau samples).

Only sequences >200 bp long with an average quality score >25 and no ambiguous characters were included in the analyses (45). Filtering of the sequences to remove sequence errors and chimeras was conducted using the USEARCH tool in QIIME (46), version 1.9.0. Phylotypes were identified using the open-frame method Uclust (47) and assigned to OTUs defined at ≥97% sequence identity. A representative sequence was chosen from each OTU by selecting the most highly connected sequence. All representative sequences were aligned by PyNAST (48). Taxonomic identity of each OTU was determined using the Greengenes database (http://greengenes.lbl.gov). To correct for survey effort, we used a randomly selected subset of 3,000 sequences per sample.

### North American samples.

Details of sample collection and bioinformatics for northern North American prokaryotes are given in references 24 and 39.

### Statistical analyses (Tibetan Plateau samples).

Correlations between diversity estimates and soil characteristics were conducted by SPSS 20.0 for Windows. Nonmetric multidimensional scaling analyses were performed using vegan of R 2.3.0 (49), based on dissimilarity calculated using the Bray-Curtis index (rarefaction depth 3,000 sequences), and these summaries of community composition were associated with environmental factors (scaled by Euclidean geographic distance between sampling sites) by using the envfit and vif of vegan package and Mantel tests.

### Historical climate data.

For assessing whether historical or current climate is more predictive of current prokaryotic distributions, we utilized global maps of monthly historical climate records from the 0.5-degree gridded CRU TS3.21 data set (34). The CRU TS3.21 data set spans 1901 to 2014, but we used only records postdating 1950, because in the Tibetan Plateau and North America, records prior to then are based on substantially more interpolation (50). We considered the following climate variables: frost day frequency, potential evapo-transpiration, daily mean temperature, monthly average daily minimum temperature, monthly average daily maximum temperature, vapor pressure, wet day frequency, cloud cover, diurnal temperature range, and precipitation (Table S1G). We considered 10- and 20-year climatologies (i.e., summaries over one or two decades) for each of these variables as predictors, but use of both climatologies yielded qualitatively similar results, so we focused on results with 10-year climatologies overlapping by 5 years (decades ending on December 31 of 2009, 2004, 1999, 1994, 1989, 1984, 1979, 1974, 1969, 1964, and 1959). Inclusion of even more time-specific climate data (e.g., from the month of sample collection) did not improve model performance. To test associations with contemporary climate, we used the average conditions from the year (January 1 to December 31) when samples were collected (2011 in Tibet and 2005 in northern North America). We performed principal-component analysis (PCA) on these variables across all climatologies and locations separately in Tibet and North America. In this PCA, each location-time period combination is an observation and each of the 10 climate measurements is a variable. These data were centered and scaled before performing the singular value decomposition step of PCA. We performed subsequent analyses using the projections of the location-time period observations onto the first three principal component axes (see Results and Discussion).

### Modeling.

To assess associations with contemporary and historic climate, we obtained from the aforementioned maps climate variables for each sampling location. Tables S2 and S3 show climate data used for modeling prokaryotic communities in the Tibetan Plateau and in northern North America, respectively, and are available at https://www.dropbox.com/sh/erekiq5l29tvqfj/AAAH9MoPB31qa6_LFmpXIKbYa?dl=0. To assess associations between contemporary prokaryotic distributions and contemporary and historic climate, we constructed regression models. We constructed separate models for the distributions of OTU richness and Shannon diversity and the relative abundance of all families (*n* = 53) and OTUs (*n* = 317) occurring in 40 or more samples. We used leave-one-out cross-validation to assess model performance and perform model selection. We performed all-subset model selection with all of the climatology dates. In the absence of any clear nonlinearity, we employed linear models to further minimize the risk of overfitting. Diversity response variables (richness and Shannon diversity) were log-transformed prior to modeling, and relative abundance response variables were logit-transformed. To assess robustness of our findings to modeling choices, we (i) repeated regression modeling with the original climatologies rather than PCs using a two-step variable selection procedure in which the top ∼5 variables were chosen using only 1960–1969 and contemporary climatologies and then all-subset model selection was performed over all time periods for these top variables (all-subsets is computationally impractical with 120 variables) and (ii) fitted gradient-boosted regression models with all PCs rather than performing all-subset model selection.

To predict how prokaryotic communities would shift if they were to equilibrate to contemporary climate, we substituted contemporary climate data (most recent 10-year climatology) into the models selected above, many of which used climate data from prior to 1980. To estimate shifts in diversity and relative abundance, we took the difference between future predictions and contemporary predictions (as opposed to the difference between future predictions and contemporary observations); this procedure avoided spurious correlations that can arise from the nonzero covariance that always exists between residuals and observed values. We used Multivariate Environmental Similarity Surface (MESS [51]) to ensure that the maps of diversity that we projected did not require excessive extrapolation (Fig. S5D and S9E).

### Code availability.

Code is available at https://github.com/jladau/SpeciesDistributionModeling.

### Data availability.

The 454 pyrosequencing data set of Tibetan soil prokaryotes is deposited in the DDBJ Sequence Read Archive (http://trace.ddbj.nig.ac.jp/DRASearch) with accession number DRA001226.
