# Supplementary figures and images for: Existing Climate Change Will Lead to Pronounced Shifts in the Diversity of Soil Prokaryotes
Source: mSystems. 2018 Oct 23;3(5):e00167-18. doi: 10.1128/mSystems.00167-18 (PMC6199470; doi:10.1128/mSystems.00167-18)

Figure S1.

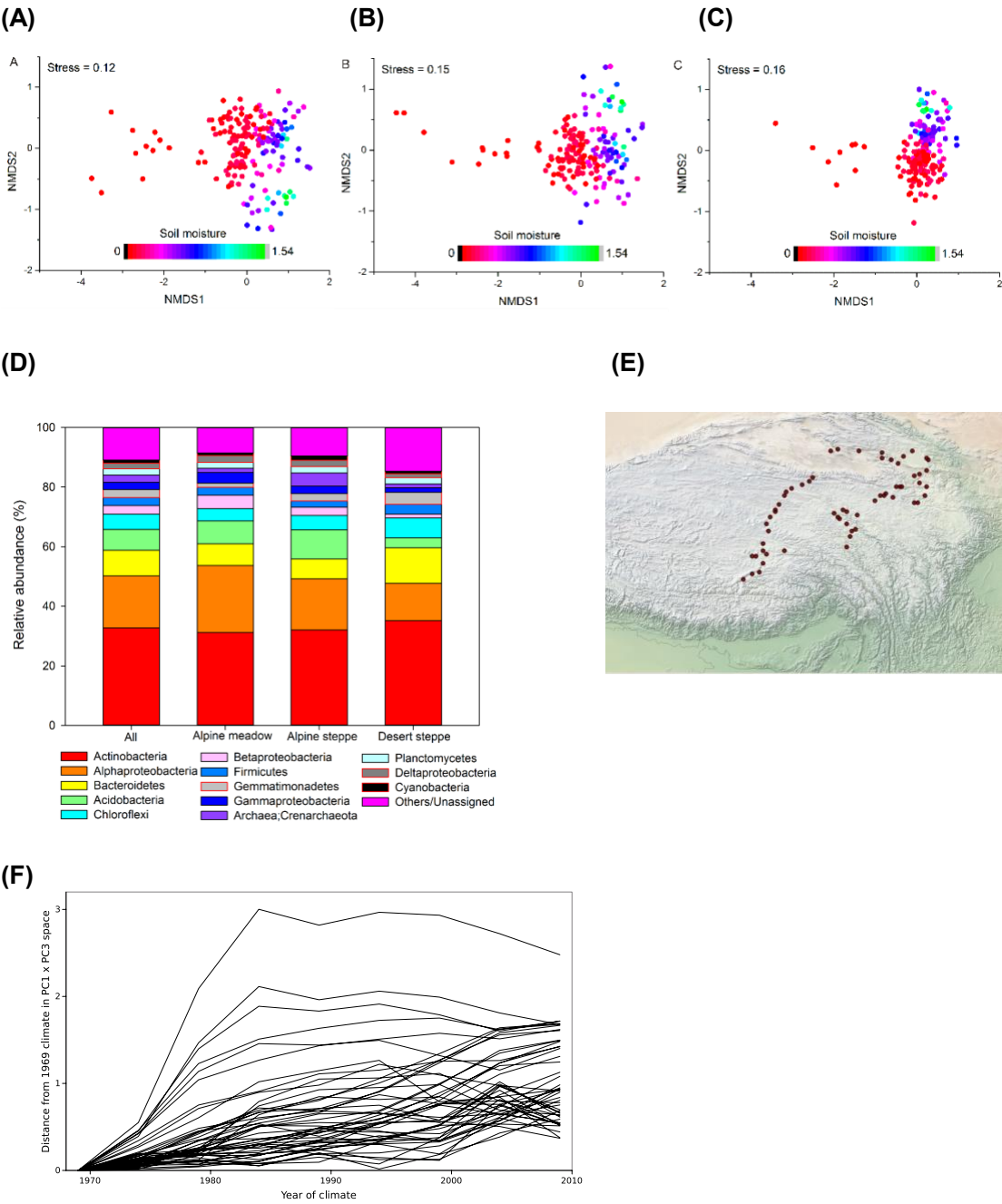

Supplement: FIG S1 [file sys005182280sf1.pdf]

**Figure S2.**

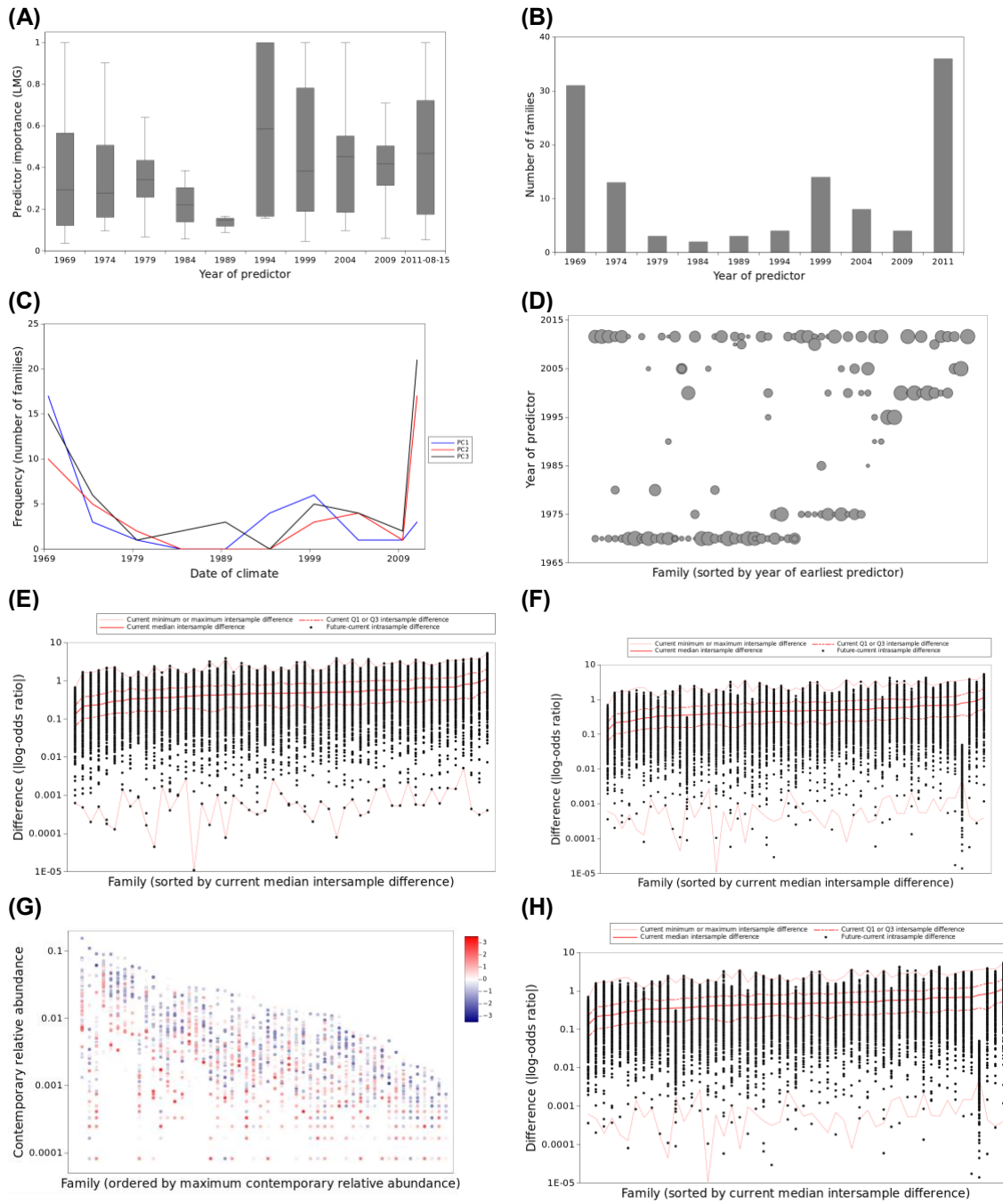

Supplement: FIG S2 [file sys005182280sf2.pdf]

Figure S3.

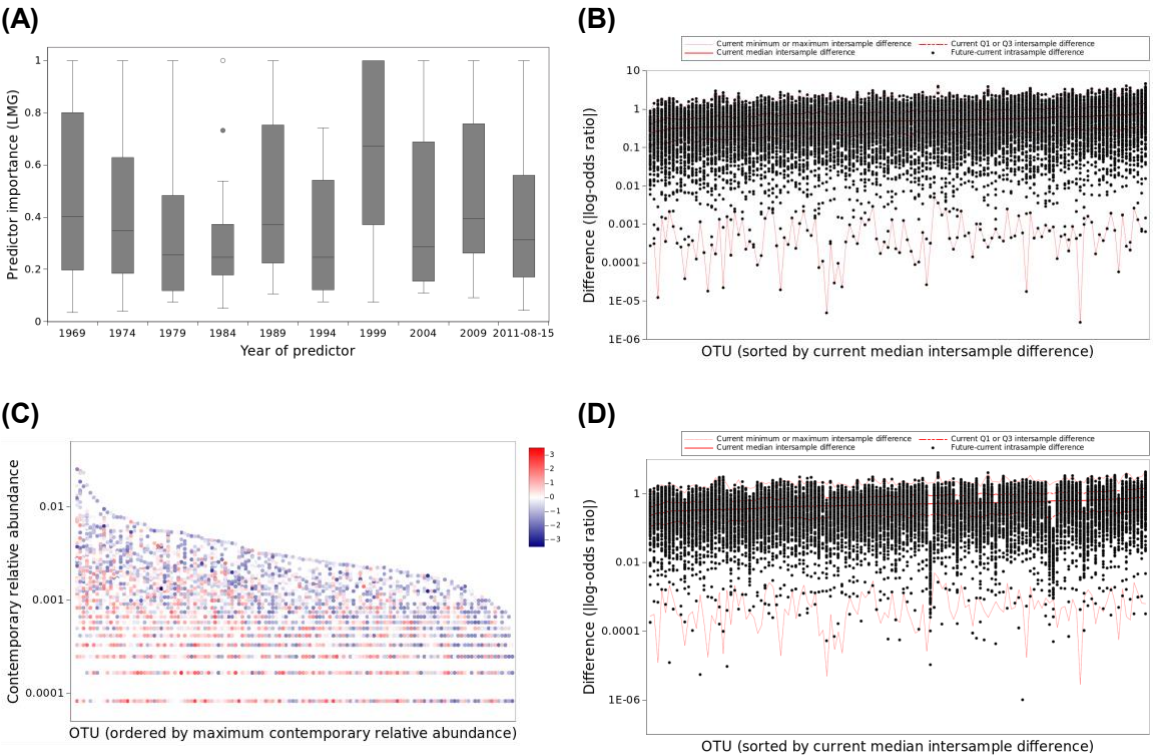

Supplement: FIG S3 [file sys005182280sf3.pdf]

Figure S4.

(A)

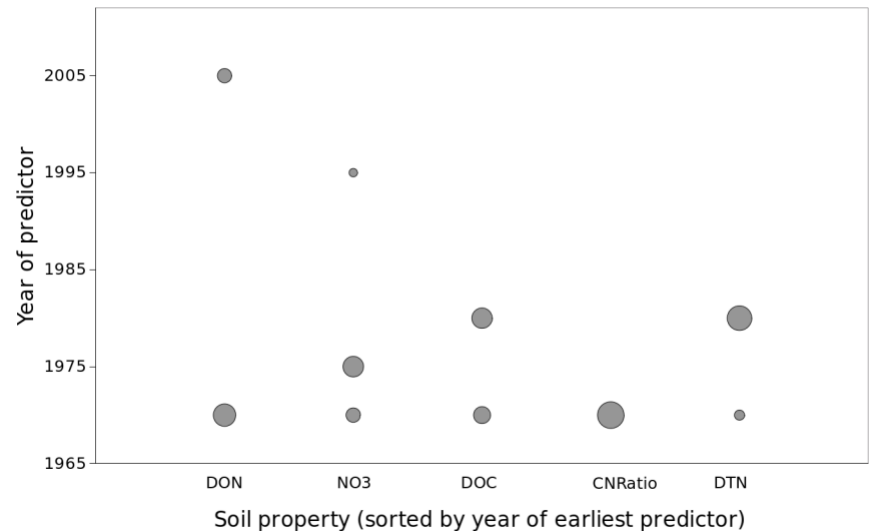

(B)

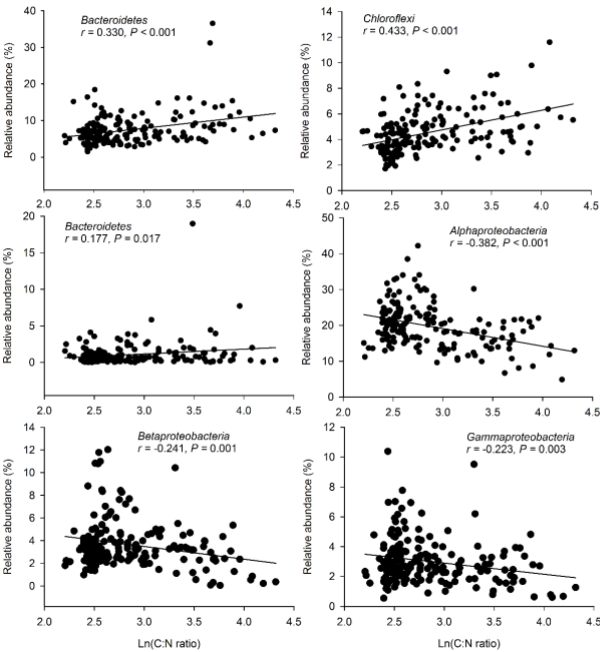

(C)

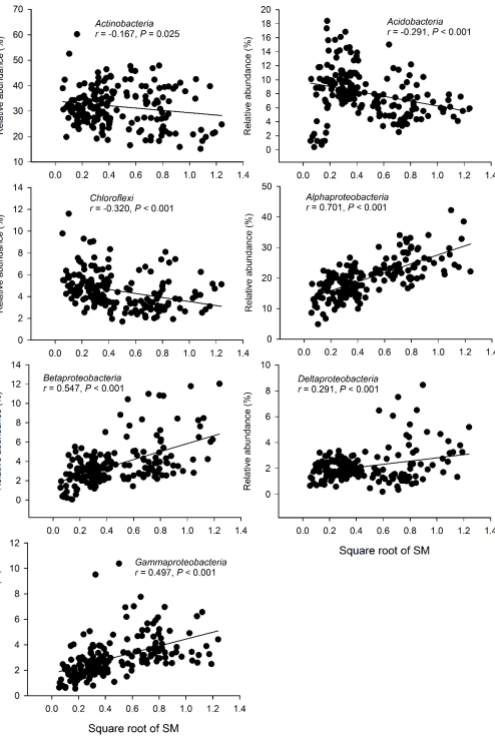

Supplement: FIG S4 [file sys005182280sf4.pdf]

Figure S5.

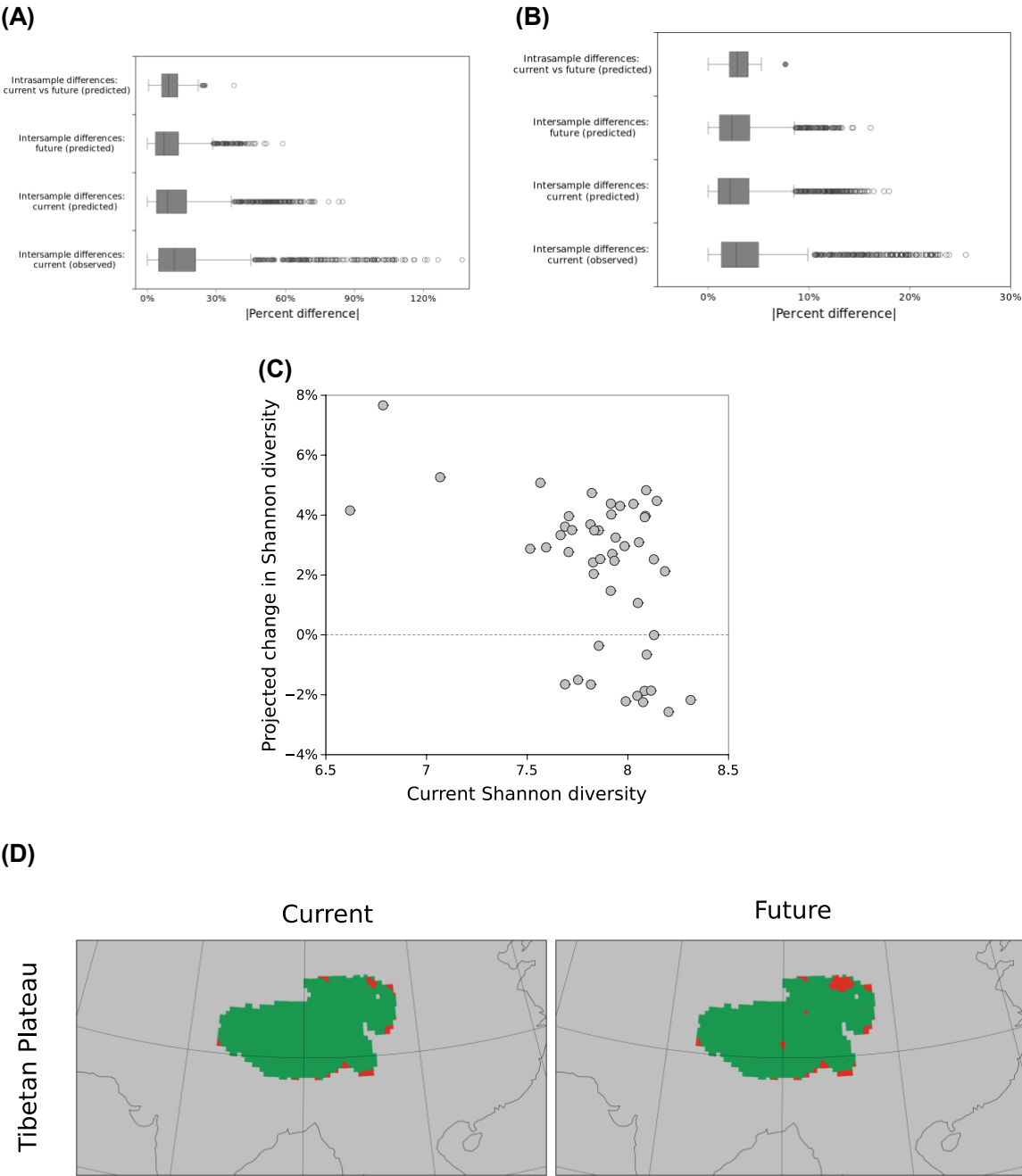

Supplement: FIG S5 [file sys005182280sf5.pdf]

Figure S6.

(A)

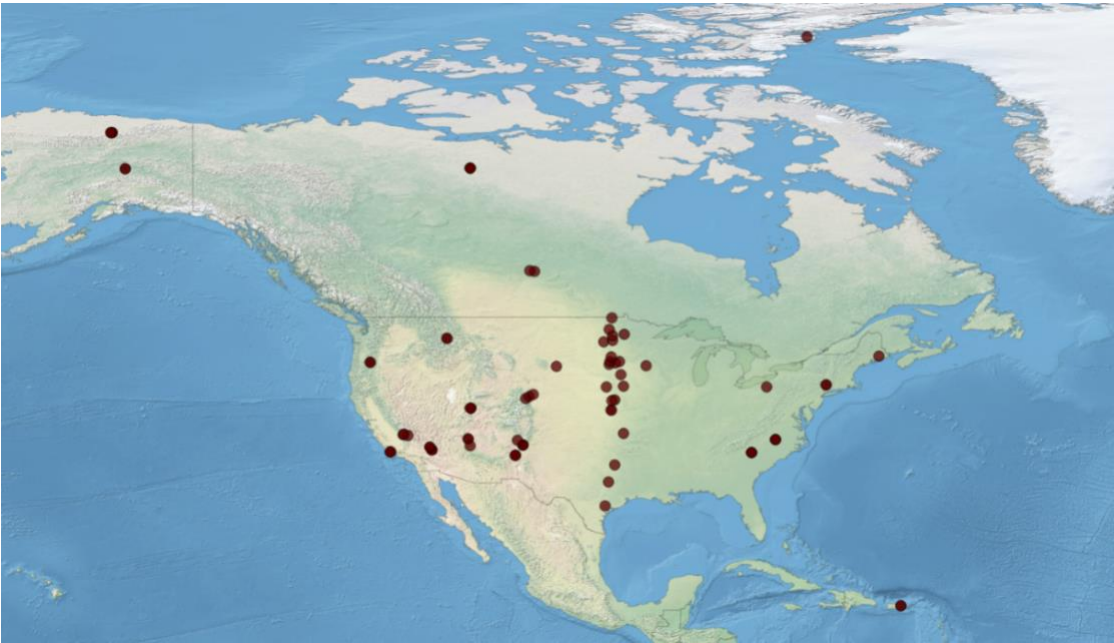

(B)

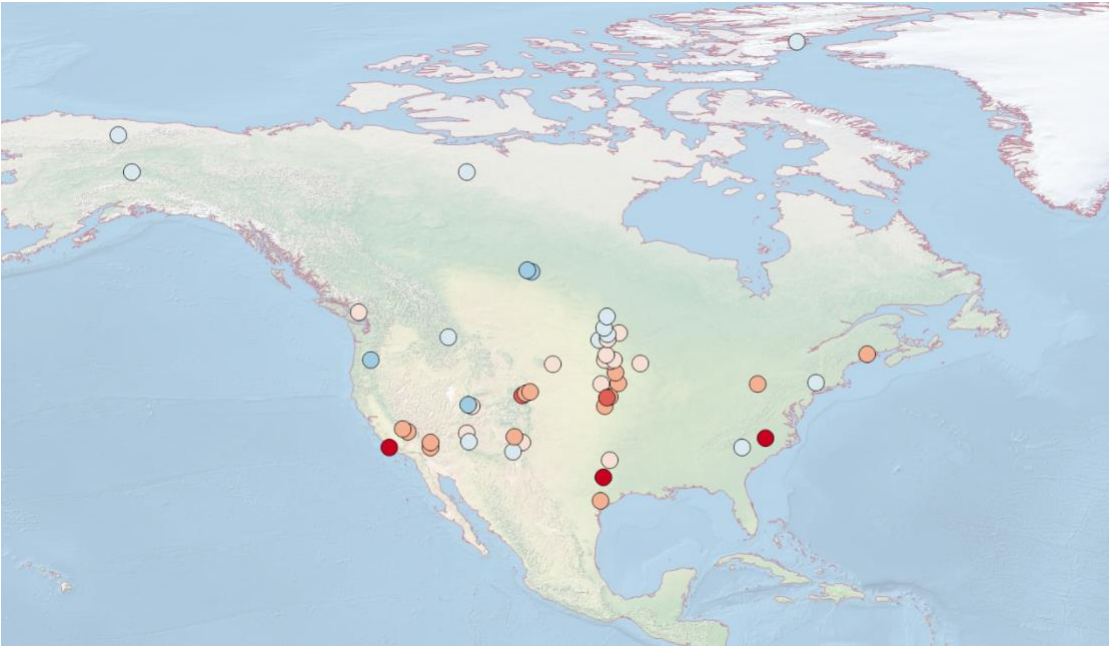

Supplement: FIG S6 [file sys005182280sf6.pdf]

**Figure S7.**

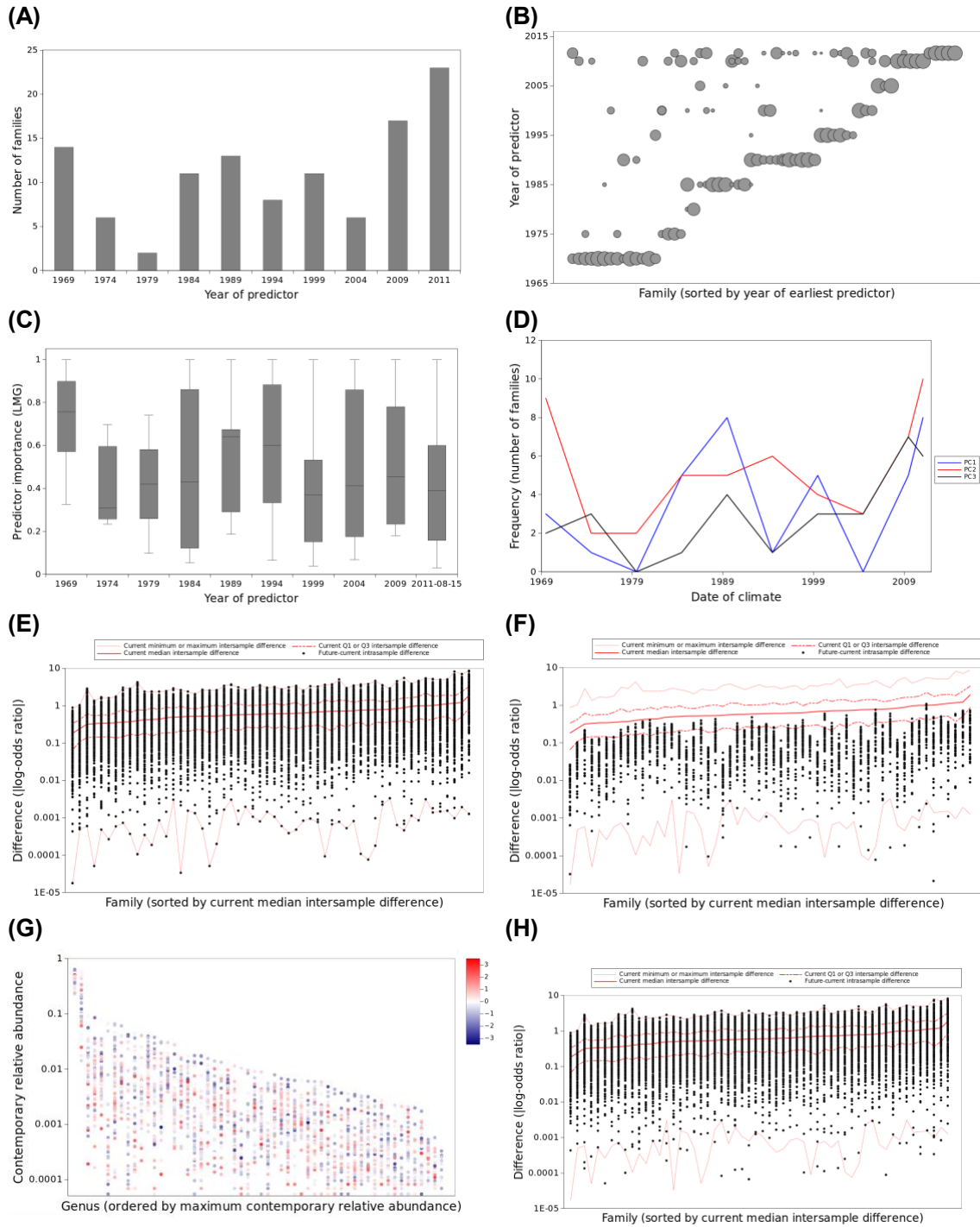

Supplement: FIG S7 [file sys005182280sf7.pdf]

**Figure S8.**

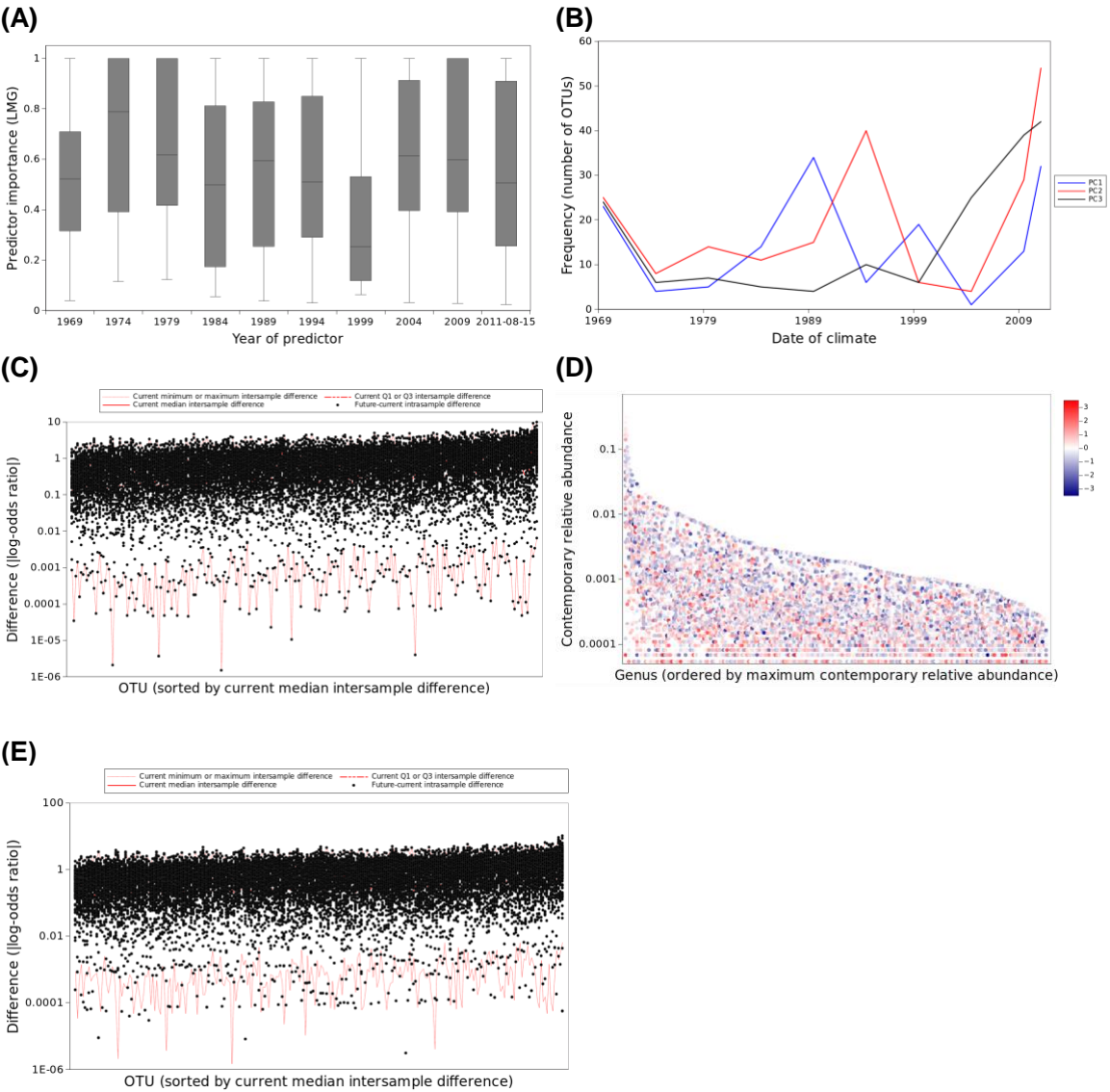

Supplement: FIG S8 [file sys005182280sf8.pdf]

**Figure S9.**

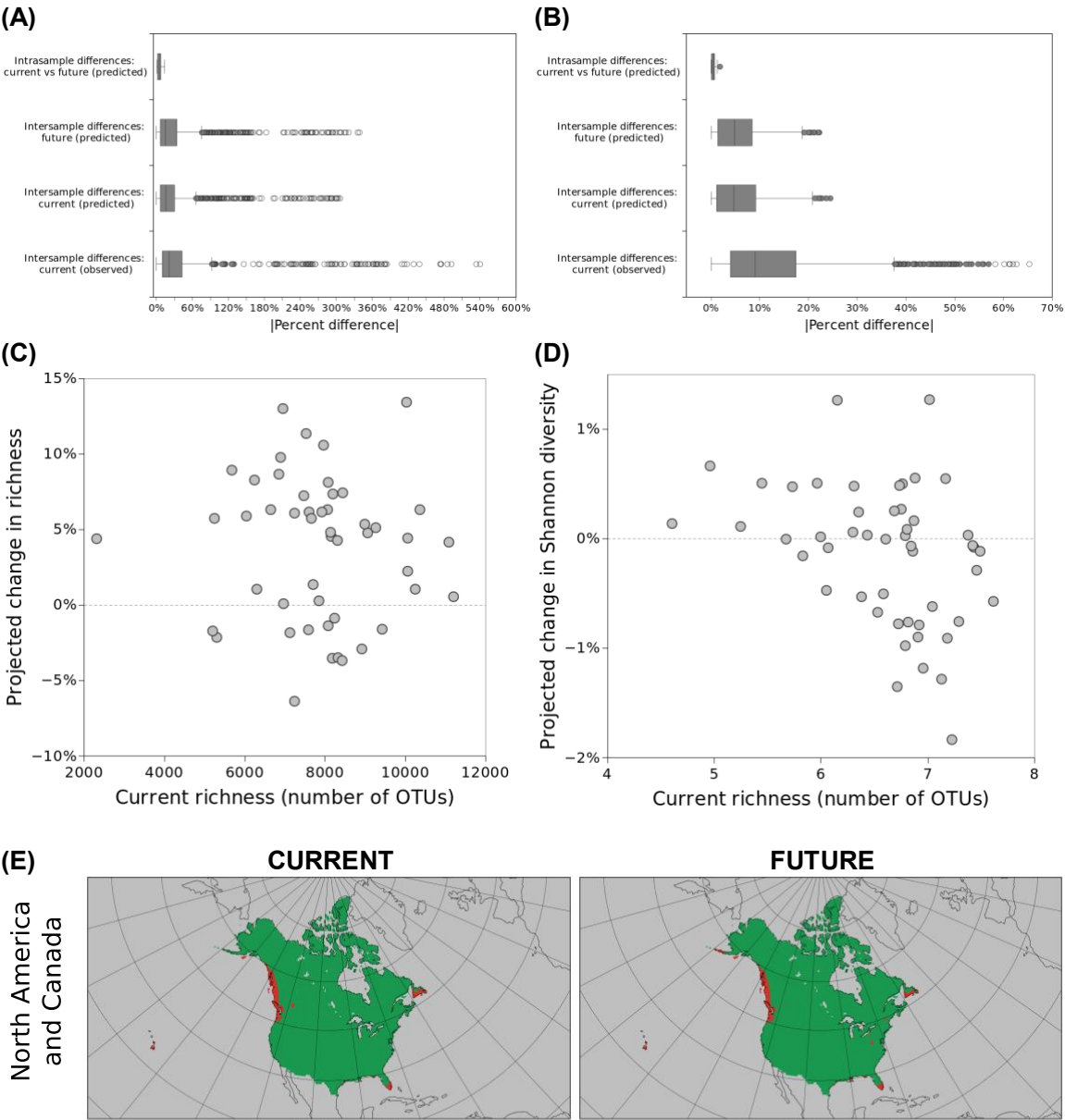

Supplement: FIG S9 [file sys005182280sf9.pdf]
